# Effectiveness and safety of glucagon-like peptide 1 receptor agonists in patients with type 2 diabetes: evidence from a retrospective real-world study

**DOI:** 10.3389/fendo.2024.1347684

**Published:** 2024-03-08

**Authors:** Yan Jiang, Han-Sheng Bai, Guo-Xin Liu, Shi-Yi Wang, Li Yin, Zhao-Ting Hou, Chen-Yang Zhao, Guang-Jun Fan

**Affiliations:** Department of Pharmacy, The Second Affiliated Hospital of Dalian Medical University, Dalian, China

**Keywords:** GLP-1RA, real-world evidence, type 2 diabetes mellitus, effectiveness, safety

## Abstract

**Introduction:**

Global phase III clinical trials have shown superior hypoglycemic efficacy to insulin and other oral hypoglycemic agents. However, there is a scarcity of real-world data comparing different glucagon-like peptide 1 receptor agonist (GLP-1RA) directly. This study aimed to assess the safety and effectiveness of various GLP-1RA in treating type 2 diabetes mellitus (T2DM) in a real-world clinical setting and identify predictive factors for favorable treatment outcomes.

**Methods:**

This was a retrospective, single-center, real-world study. The changes in HbA1c, fasting plasma glucose (FPG), body weight, systolic blood pressure (SBP), diastolic blood pressure (DBP), and the percentage of participants who achieved HbA1c of <7%, 7%-8%, and ≥ 8% after GLP-1RA treatment was analyzed. The clinical factors that affect the effectiveness of GLP-1RA were analyzed.

**Results:**

At baseline, the 249 participants had a mean baseline HbA1c of 8.7 ± 1.1%. After at least three months of follow-up, the change in HbA1c was -0.89 ± 1.3% from baseline. Dulaglutide exerted a more significant hypoglycemic effect than immediate-release exenatide. The percentage of participants who achieved HbA1c<7% was substantial, from 6.0% at baseline to 28.9%. Average body weight decreased by 2.02 ± 3.8 kg compared to baseline. After GLP-1RA treatment, the reduction in SBP was 2.4 ± 7.1 mmHg from baseline. A shorter duration of diabetes and a higher baseline HbA1c level were more likely to achieve a good response in blood glucose reduction.

**Conclusions:**

This study provided real-world evidence showing that GLP-1RA significantly improved HbA1c, body weight, and SBP. The results can inform the decision-making about GLP-1RA treatment in daily clinical practice.

## Introduction

Type 2 diabetes mellitus (T2DM), one of the chronic metabolic disorders, causes a disorder in insulin secretion, leading to hyperglycemia. The pathophysiology of T2DM involves a combination of two main factors: decreased insulin secretion by pancreatic β-cells and the ability of insulin-sensitive tissues to respond appropriately to insulin. In the past decades, new hypoglycemic drugs have been continuously explored and have been administered to patients with T2DM, including dipeptidyl peptidase-4 inhibitors (DPP-4i), glucagon-like peptide 1 receptor agonists (GLP-1RA), and sodium-glucose cotransporter 2 inhibitors (SGLT-2i) (1). GLP-1, a gut-derived peptide, is excreted from intestinal epithelial L-cells, which enhance pancreatic β-cells to secrete insulin in the body (2). Compared to other hypoglycemic drugs, especially insulin, GLP-1RA produces more robust glucose-lowering effects, weight reduction, and lower risk of hypoglycemia (3). Furthermore, some long-term observational studies have shown that GLP-1RA plays a vital role in cardiovascular, renal, and stroke protection, contributing to the reduction of major adverse cardiovascular events, the delay of the progression of diabetic nephropathy, and the reduction of mortality in patients with T2DM (4–7).

The pharmacological action of GLP-1RA is based on the stimulation of glucose-dependent insulin secretion, glucagon secretion reduction, and slowing gastric emptying, resulting in improved glycemic control and modest weight loss with a reduced risk of hypoglycemia (8). Consequently, the main side effect of GLP-1RA is gastrointestinal disorders, including nausea, vomiting, and diarrhea (9). To reduce the incidence of adverse events and improve patient tolerance, longer-acting GLP-1RA has been widely administered in patients with T2DM (10). Based on their distinct molecular structures, dosage forms, and pharmacokinetics, the dosage regimens of various GLP-1RA vary, ranging from twice daily (exenatide immediate-release, IR) to once daily (liraglutide and lixisenatide) and once weekly (exenatide extended-release, loxenatide, dulaglutide, and semaglutide) (11). Despite this, the phase III clinical trials of GLP-1RA, such as AWARD-6 and SUSTAIN-7, still indicated that more than 60% of study participants had treatment-emergent adverse event and approximately 3% discontinued treatment prematurely due to a gastrointestinal adverse event, with nausea being the most common reason (12, 13).

However, these sufficient evidences were primarily derived from randomized controlled trials (RCT) rather than real-world studies (RWS). RCT was considered as the gold standard for evaluating drug efficacy and safety, but the results remained influenced by sampling variability and potential bias from non-adherence and loss of information (14). RWS is considered to complement and improve the results of RCT, most of which are observational studies, to reflect the effectiveness and safety of therapeutic agents in clinical practice (15, 16). Here, we performed a retrospective RWS to evaluate the effectiveness and safety of GLP-1RA in treating T2DM in routine clinical practice, providing objective evidence for guiding clinicians to use GLP-1RA.

## Methods

### Study design and participants

This retrospective RWS was conducted at the Second Affiliated Hospital of Dalian Medical University from January 2015 to June 2022. The study enrolled participants with T2DM who were treated with GLP-1RA. The decision to initiate GLP-1RA therapy was at the treating physician’s discretion. Participants who met the following criteria were included: (1) be 18 years or older, (2) had a documented diagnosis of T2DM at least 12 weeks before enrollment, and (3) had at least one recorded HbA1c value within 12 weeks before providing informed consent and beginning treatment. Participants were excluded if they met any of the following criteria: (1) pregnancy, (2) an estimated glomerular filtration rate (eGFR) of ≤15 mL/min/1.73m^2^, or (3) had no documented HbA1c value within six months after providing consent and starting treatment.

### Data extraction

The baseline date was the date of initiation of GLP-1RA treatment. Clinical characteristics and laboratory data were collected within 90 days before this baseline date. These included age, sex, duration of diabetes, body height and weight, body mass index (BMI), systolic and diastolic blood pressure (SBP and DBP), HbA1c, fasting plasma glucose (FPG), total cholesterol (TC), triglyceride (TG), high density lipoprotein cholesterol (HDL), low density lipoprotein cholesterol (LDL), alanine aminotransferase (ALT), aspartate transaminase (AST), serum creatinine (SrCr), and eGFR. Furthermore, information on concomitant medication was recorded. The study identified participants with subsequent hospital visits within 12 months of the baseline data collection. HbA1c, FPG, SBP, DBP, and body weight were collected during these visits.

### Study endpoints

The study compared the changes observed in glycemic parameters (HbA1c and FPG) and non-glycemic parameters (body weight, SBP, and DBP) in participants treated with GLP-1RA. Primary endpoints were the HbA1c (%) and FPG (mmol/L) changes from the baseline to the follow-up period. Additionally, the study assessed the number of individuals categorized as HbA1c<7%, 7%≤HbA1c<8%, and HbA1c≥8% at the follow-up (17). Secondary endpoints included changes in body weight, SBP, and DBP from baseline to follow-up and the occurrence of adverse effects.

### Statistical analysis

Continuous variables are expressed as mean ± standard deviation (SD), while categorical variables are presented as numbers and percentages. To compare the changes in each parameter before and after using GLP-1RA, paired t-tests were employed for continuous variables. For parameters with three or more groups of participants, one-way analysis of variance (ANOVA) or Kruskal-Wallis tests were used for comparison (18). A logistic regression analysis was performed to investigate the impact of various clinical factors on GLP-1RA with a good-response. We categorized participants into two groups based on their response to treatment: the good-response group, defined as those who demonstrated a change in HbA1c greater than or equal to the median (≥0.8%), and the poor-response group, defined as those who showed a change in HbA1c less than the median (<0.8%). The participants were divided into different groups based on age (younger group: age <65 years, elderly group: age ≥65 years), obesity status (non-obese group: BMI <28 kg/m^2^, obese group: BMI ≥28 kg/m^2^), and sex. Baseline HbA1c levels were categorized as <8.0% or ≥8.0%. The duration of diabetes was divided into <10 or ≥10 years using the respective medians. *P*<0.05 was considered statistically significant. Data analysis was performed using SPSS Statistics (version 27).

## Results

### Patient demographics and characteristics

A total of 249 participants were analyzed: exenatide-IR (n=50), liraglutide (n=50), loxenatide (n=50), dulaglutide (n=50), and lixisenatide (n=49). **Table 1** shows the baseline characteristics of these participants. The therapeutic maintenance dose for each GLP-1RA was 20 μg/day for exenatide-IR, 1.8 mg/day for liraglutide, 20 μg/day for lixisenatide, 1.5 mg/week for dulaglutide, and 0.2 mg/week for loxenatide. The average age of the participants was 56.9 ± 11.8 years, and 133 (53.4%) were men. The mean duration of diabetes was 10.5 ± 7.2 years. The baseline BMI was 28.4 ± 2.9 kg/m^2^, and the HbA1c level was 8.7 ± 1.1%. The prevalence of microangiopathy and macroangiopathy was 124 (49.8%) and 189 (75.9%), respectively. The most commonly used concomitant diabetes medications were insulin (130, 52.2%) and glycosidase inhibitors (104, 41.8%). The mean length of follow-up was 9 ± 2 months.

**Table 1:** Clinical baseline characteristics of the participants.

Variables	
Number of participants, n	249
Men/women, n/n	133/116
Age, years	56.9 ± 11.8
Duration of diabetes, years	10.5 ± 7.2
BMI, kg/m^2^	28.4 ± 2.9
Weight, kg	80.3 ± 12.0
HbA1c, %	8.7 ± 1.1
HbA1c <7%, n	15
7%≤ HbA1c <8%, n	68
HbA1c ≥8%, n	166
SBP, mmHg	142.7 ± 18.0
DBP, mmHg	81.8 ± 9.8
FPG, mmol/L	9.7 ± 2.7
TC, mmol/L	5.1 ± 1.5
TG, mmol/L	2.1 ± 1.0
LDL-C, mmol/L	2.6 ± 1.0
HDL-C, mmol/L	1.1 ± 0.3
AST, U/L	25.0 ± 15.8
ALT, U/L	33.0 ± 34.9
eGFR, mL/min/1.73 m^2^	101.2 ± 27.3
Complications	
Microangiopathy, n	124
Macroangiopathy, n	189
Neuropathy, n	188
Diabetes medications	
Metformin, n	98
Secretagogue, n	55
Glycosidase inhibitors, n	104
TZD, n	19
DPP-4i, n	52
SGLT-2i, n	92
Insulin, n	130
Insulin dose, IU	28.1 ± 14.8
Other medications	
ACEi/ARB, n	72
CCB, n	89
β- blocker, n	31
Diuretics, n	4
Statin, n	137
Fibrate, n	17
APT, n	75
Prescription of GLP-1RAs	
Exenatide-IR, n	50
Liraglutide, n	50
Lixisenatide, n	49
Dulaglutide, n	50
Loxenatide, n	50
Follow-up (months)	9.0 ± 2.0

Data are presented as n for categorical variables and as the mean ± SD for continuous variables.

BMI, body mass index; SBP, systolic blood pressure; DBP, diastolic blood pressure; FPG, fasting plasma glucose; TC, total cholesterol; TG, triglycerides; LDL, low density lipoprotein; HDL, high density lipoprotein; AST, aspartic aminotransferase; ALT, alanine aminotransferase; eGFR, estimated glomerular ﬁltration rate; TZD, Thiazolidinedione; DPP-4i, dipeptidyl peptidse 4 inhibitor; SGLT-2i, sodium-dependent glucose transporters 2 inhibitor; ACEi, angiotensin converting enzyme inhibitors; ARBs, angiotensin receptor blockers; CCB, calcium channel blockers; APT, anti-platelet therapies; GLP-1RA, glucagon-like peptide 1 receptor agonists; Exenatide-IR, exenatide immediate-release.

### The primary endpoint of HbA1c and response predictors

**Figure 1:**
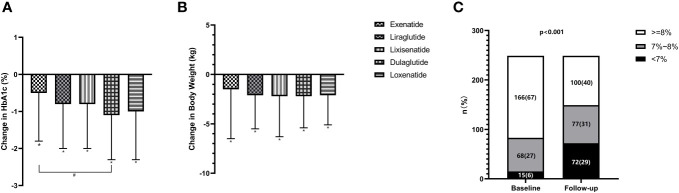
Change in HbA1c and body weight from baseline in GLP-1RAs subgroup **(A, B)** and proportion of participants achieving HbA1c<7% **(C)**. *P<0.05 in the paired t-test (vs. baseline). #*P*<0.05 in the independent t-test (exenatide-IR vs. dulaglutide).

Participants treated with GLP-1RA had a mean reduction in HbA1c levels of 0.89 ± 1.3% from baseline. HbA1c values in the five treatment groups improved significantly compared to their baseline values. Among the treatments, dulaglutide had the greatest reduction in HbA1c of 1.06% [95% confidence interval (CI) -1.40 to -0.72%, *P*<0.001], followed by loxenatide (-0.98%; 95% CI -1.35 to -0.61%, *P*<0.001), liraglutide (-0.82%; 95% CI -1.16 to -0.48%, *P*<0.001), lixisenatide (-0.76%; 95% CI -1.12 to -0.41%, *P*<0.001), and exenatide-IR (-0.54%; 95% CI -0.92 to -0.16%, *P*<0.001). Details are shown in **Supplementary Table 2**. Further analysis revealed a statistically significant difference in HbA1c changes between dulaglutide and exenatide-IR groups (*P*=0.042). However, no significant differences were observed between the other two groups (**Figure 1**).

The percentage of participants who achieved HbA1c levels below 7% increased significantly (*P*<0.001) from 6.0% at baseline to 28.9% after treatment. Similarly, there was a significant decrease in the percentage of participants with HbA1c levels equal to or greater than 8% in all treatment groups (*P*<0.001), decreasing from 66.7% at baseline to 40.2%. However, no significant differences were observed in the distribution of HbA1c levels among the five treatment groups. Details are shown in **Figure 1**.

Logistic regression analysis was conducted to identify potential predictors of good response to GLP-1RA treatment (**Table 2**). A shorter duration of T2DM (*P*=0.037) and a higher baseline HbA1c level (*P*<0.001) were more likely to be associated with achieving a good response than other predictors.

**Table 2:** Independent clinical factors associated with good response.

Parameters	n(%)	OR	95% CI	*P* value
Age < 65 years	177(71.1%)	1.716	0.916-3.217	0.092
Sex, female	116(46.6%)	1.036	0.603-1.779	0.899
BMI < 28 kg/m^2^	118(47.4%)	1.163	0.675-2.003	0.587
Diabetes duration < 10 years	126(50.6%)	1.808	1.038-3.150	0.037
Baseline HbA1c < 8.0%	83(33.7%)	0.245	0.136-0.443	<0.001

BMI, body mass index; OR, odds ratio; CI, confidence interval.

### The primary endpoint of fasting plasma glucose

GLP-1RA treatment was associated with a relatively significant decrease in FPG levels, 0.50 ± 3.4 mmol/L from baseline. All five treatment groups showed a decline in FPG levels compared to their baseline values. The dulaglutide group had the highest reduction in FPG levels (-0.95 mmol/L; 95% CI -0.06 to -1.84) followed by liraglutide (-0.67 mmol/L; 95% CI 0.18 to -1.52%), lixisenatide (-0.36 mmol/L; 95% CI 0.58 to -1.30%), exenatide-IR (-0.35 mmol/L; 95% CI -1.40 to -0.97%), and loxenatide (-0.16 mmol/L; 95% CI 0.66 to -1.16%). However, statistically significant differences were observed only in the dulaglutide group (*P*=0.037). Additionally, there were no statistically significant differences between the two groups regarding FPG reduction. Details are shown in **Supplementary Table 2**.

### Secondary endpoints of body weight

The mean body weight was decreased by 2.02 ± 3.8 kg from baseline. When comparing the baseline body weight values between the five treatment groups, the dulaglutide group had the highest baseline weight (80.67 ± 10.9 kg) and the most significant change in body weight (-2.23 kg; 95% CI -1.33 to -3.13, *P*<0.001). Over time, the average body weight decreased in all five groups, and significant differences were observed (**Figure 1**). Unfortunately, no statistically significant differences were found between any two specific groups. Consistent with changes in body weight, a reduction in mean BMI was also observed after GLP-1RA treatment.

### Secondary endpoints of blood pressure

After GLP-1RA treatment, there was a reduction of 2.37 ± 7.1 mmHg in SBP from baseline. When comparing the SBP values with their respective baselines, the five treatment groups showed a decrease. The dulaglutide group showed the highest decrease in DBP (-3.16 mmHg; 95% CI -1.31 to -5.01%, *P*<0.001), followed by the loxenatide group (-2.96 mmHg; 95% CI -1.55 to -4.37%, *P*<0.001), liraglutide (-2.38 mmol/L; 95% CI -0.54 to -4.22%, *P*=0.012), lixisenatide (-2.00 mmol/L; 95% CI -0.40 to -3.60%, *P*=0.016), and the exenatide-IR group (-1.36 mmHg; 95% CI 1.71 to -4.43%). However, only the exenatide-IR group showed no significant difference (*P*=0.377). Additionally, there were no statistically significant differences between the two groups regarding SBP reduction.

Among the different drugs, the loxenatide and liraglutide groups showed an increase in DBP of 0.38 ± 5.7 mmHg and 0.24 ± 7.2 mmHg, respectively. While the dulaglutide, lixisenatide, and exenatide-IR groups showed a decrease in DBP of 1.00 ± 6.0 mmHg, 0.47 ± 8.0 mmHg, and 0.42 ± 7.1 mmHg, respectively. These changes were not statistically significant.

### The secondary endpoint of adverse events

Three participants (1.2%) reported adverse events (1 in the exenatide-IR group and 2 in the dulaglutide group). All were gastrointestinal reactions. Among the three participants, one in the dulaglutide group discontinued the medication after seven months of treatment.

## Discussion

The present study compared the effectiveness and safety of GLP-1RA (exenatide-IR, liraglutide, lixisenatide, loxenatide, and dulaglutide) in Chinese participants with T2DM in a real-world setting. Participants treated with GLP-1RA showed significant reductions in HbA1c, body weight, FPG, and SBP. Dulaglutide significantly reduced HbA1c more than exenatide-IR. However, no significant differences in HbA1c reduction between dulaglutide and the other drugs were observed. The incidence of adverse reactions to GLP-1RA was extremely low, indicating that GLP-1RA was well tolerated in Chinese participants. The hypoglycemic effect of GLP-1RA was more remarkable in participants with a higher baseline HbA1c (≥ 8.0%) and a shorter duration of diabetes (< 10 years).

First discovered in the early 1980s, GLP-1 was regarded as a pro-glucagon decomposition product produced in intestinal L cells (19). Further research suggested that GLP-1 can increase β-cell mass by increasing cellular regeneration, inhibiting apoptosis, and stimulating insulin biosynthesis and secretion (20–22). Extensive efforts have been made to use the glucose-lowering effects GLP-1 to treat T2DM. However, due to the fast metabolism *in vivo* and the need for subcutaneous administration, GLP-1 has been limited in therapeutic applications. Compared to endogenous GLP-1, GLP-1RA exhibited a longer half-life after subcutaneous administration (23). Furthermore, oral GLP-1RA has been applied in clinical practice to treat T2DM (24). These pharmacological strategies improved patient adherence, allowing them more options to lower blood glucose levels. Different types of GLP-1RA have significant differences in their ability to reduce HbA1c, such as exenatide-IR (0.80%), lixisenatide (0.36%), dulaglutide (1.33-1.73%), loxenatide (1.5%), and liraglutide (2.87%) (25–29). Similarly, our study showed that dulaglutide provided more excellent glycemic control than exenatide-IR, which confirmed the previous findings. More significant and more consistent reductions in HbA1c levels were observed with GLP-1RA with longer action compared to short-acting agents (30–32).

Treatment adherence, defined as the ratio of days covered by a GLP-1RA prescription to the total days in the measurement period (33), is essential to achieving glycemic control in patients with T2DM. Adherence was influenced by various factors such as side effects, costs, and cognition aspects. However, the complexity of the treatment strategy was regarded as a pivotal determinant of adherence. Previous real-world evidence from researches (34, 35) identified a notably higher adherence rate among individuals receiving long-acting GLP-1RA compared to those employing short-acting GLP-1RA therapies. Furthermore, the studies have shown that adherent patients experienced a greater reduction in HbA1c levels in comparison to non-adherent patients (36). Compared to exenatide-IR, dulaglutide extended the dosing interval to relieve the panic caused by subcutaneous injections resulting in enhance adherence, which should accounted for our research’s findings.

GLP-1RA combined with some hypoglycemic medications such as metformin and DPP-4i, which exhibited a synergistic effect on decreasing blood glucose levels. A randomized, placebo-controlled, double-blind trial revealed that metformin enhanced GLP-1 secretion in T2DM patients (37). The primary mechanism of action involved metformin stimulating intestinal epithelial L-cells to excrete GLP-1 and reducing GLP-1 degradation by modulating the activity of AMP-activated protein kinase (AMPK) (38, 39). Additionally, it may be a potential mechanism that metformin had the capability to modulate the gut microbiota that influenced the secretion levels of GLP-1 (40, 41). On the other hand, DPP-4i suppressed the activity of DPP-4 enzyme to decrease degradation of GLP-1 (42). Therefore, it is crucial that ensuring an evenly distributed population of patients taking metformin and DPP-4i when evaluating the hypoglycemic effects of GLP-1RA. In our study, the patients treated with metformin and DPP-4i were uniform distributed among the groups and there were no statistical differences (**Supplementary Table 1**), which contributed to the accurate assessment of the hypoglycemic effects of GLP-1RA and ensured the reliability of our results.

GLP-1RA also reduced body weight in patients with inadequately controlled T2DM, which was related to the activity of GLP-1 on central and peripheral receptors in the brain and stomach (43–45). Through suppression of appetite, delay in gastric emptying, and inhibition of gastric peristalsis, GLP-1RA treatment has been shown to improve feelings of fullness, reduce hunger, and help control energy intake, leading to a reduction in body weight (46, 47). Compared to short-acting compounds, long-acting GLP-1RA does not appear to substantially affect gastric peristalsis and emptying when administered long-term (48). Our findings indicated that regardless of whether short-acting or long-acting GLP-1RA was used, patients with T2DM experienced a significant decrease in body weight compared to baseline. However, we did not observe significant differences in the change in body weight between the different treatment groups.

Adverse reactions such as nausea, diarrhea, and vomiting, commonly associated with GLP-1RA use, can contribute to weight loss. Additionally, subcutaneous administration of GLP-1RA can rarely result in anaphylaxis, characterized by symptoms such as urticaria, pruritus, and dyspnea (49, 50). The incidence of adverse events may vary among different types of GLP-1RA due to differences in amino acid sequences. In our study and other real-world investigations, patients demonstrated good tolerance to adverse events, and such events rarely led to premature discontinuation of treatment (18, 51, 52). This can be attributed to two factors. First, most patients opt for GLP-1RA with higher homology and lower immunogenicity, such as liraglutide, semaglutide, or dulaglutide. Second, gastrointestinal adverse reactions gradually decrease or disappear progressively throughout treatment.

This RWS has several limitations. First, it was a single-center retrospective observational study that did not include a control group. The design can introduce confounding factors that were not accounted for. Therefore, the results may not apply to the larger population of individuals with diabetes in China. Second, data collection relied on a retrospective assessment. The absence of mandatory assessments at pre-specified time points could have impacted the robustness and completeness of the data. Furthermore, the average duration of follow-up, although similar to that of phase III randomized controlled trials, was relatively short. This limited duration of follow-up may not capture the long-term effects or potential changes in the treatment response over time. Lastly, it is essential to note that observational studies, such as this one, provide moderate evidence. More research is needed, including RCTs with larger sample sizes, to validate the study findings.

## Conclusions

This study demonstrates the significant benefits of GLP-1RA in improving HbA1c, body weight, and SBP in patients with T2DM. Dulaglutide exhibited superior glycemic effects compared to exenatide-IR. Patients with higher baseline HbA1c levels and shorter duration of diabetes may experience a more pronounced response to GLP-1RA. These findings have important implications for clinical decision-making when considering GLP-1RA as a treatment option for patients with T2DM.

## Data availability statement

The original contributions presented in the study are included in the article/**Supplementary Material**. Further inquiries can be directed to the corresponding authors.

## Ethics statement

The studies involving humans were approved by The Second Affiliated Hospital of Dalian Medical University. The studies were conducted in accordance with the local legislation and institutional requirements. The human samples used in this study were acquired from primarily isolated as part of your previous study for which ethical approval was obtained. Written informed consent for participation was not required from the participants or the participants’ legal guardians/next of kin in accordance with the national legislation and institutional requirements.

## Author contributions

YJ: Writing – original draft. H-SB: Writing – review & editing. G-XL: Software, Writing – review & editing. S-YW: Data curation, Investigation, Writing – review & editing. LY: Formal analysis, Investigation, Supervision, Writing – review & editing. Z-TH: Conceptualization, Data curation, Investigation, Writing – review & editing. C-YZ: Project administration, Writing – original draft. G-JF: Funding acquisition, Writing – original draft.
