# Supplementary material for: Effectiveness and safety of glucagon-like peptide 1 receptor agonists in patients with type 2 diabetes: evidence from a retrospective real-world study
Source: Front Endocrinol (Lausanne). 2024 Mar 8;15:1347684. doi: 10.3389/fendo.2024.1347684 (PMC10958196; doi:10.3389/fendo.2024.1347684)
Supplement: Supplementary file 1 [file Table_1.docx]

Table S1 Distribution of patients treating with metformin and DPP-4i in baseline

|  | Total (N) | Exenatide-IR | Liraglutide | Lixisenatide | Dulaglutide | Loxenatide | *P* |
| --- | --- | --- | --- | --- | --- | --- | --- |
| Metformin | 98 | 18/50 | 24/50 | 23/49 | 19/50 | 14/50 | 0.221 |
| DPP-4i | 52 | 13/50 | 9/50 | 10/49 | 8/50 | 12/50 | 0.724 |
