# Supplementary material for: Effectiveness and safety of glucagon-like peptide 1 receptor agonists in patients with type 2 diabetes: evidence from a retrospective real-world study
Source: Front Endocrinol (Lausanne). 2024 Mar 8;15:1347684. doi: 10.3389/fendo.2024.1347684 (PMC10958196; doi:10.3389/fendo.2024.1347684)
Supplement: Supplementary file 2 [file Table_2.pdf]

Supplementary Table 2 Change in study parameters during the follow-up period

	Exenatide-IR	N=50			Liraglutide	N=50			Lixisenatide	N=49			Dulaglutide	N=50			Loxenatide	N=50			Total	N=249		
	Baseline	Follow-up	Change	*P*	Baseline	Follow-up	Change	*P*	Baseline	Follow-up	Change	*P*	Baseline	Follow-up	Change	*P*	Baseline	Follow-up	Change	*P*	Baseline	Follow-up	Change	*P*
HbA1c, %	8.7±1.1	8.1±1.4	-0.5±1.3	0.006	8.7±1.2	8.1±1.2	-0.8±1.2	<0.001	8.6±1.2	7.9±1.4	-0.8±1.2	<0.001	8.6±1.2	7.6±1.3	-1.1±1.2	<0.001	8.7±1.1	7.7±1.4	-1.0±1.3	<0.001	8.7±1.1	7.8±1.4	-0.8±1.3	<0.001
<7%	3(6%)	9(18%)		<0.001	3(6%)	15(30%)		<0.001	3(6.1%)	13(26.6%)		<0.001	3(6%)	17(34%)		<0.001	3(6%)	18(36%)		<0.001	15(6%)	72(28.9%)		<0.001
7%~8%	12(24%)	18(36%)			14(28%)	15(30%)			13(26.5%)	18(36.7%)			16(32%)	15(30%)			13(26%)	11(22%)			68(27.3%)	77(30.9%)		
≥8%	35(70%)	23(46%)			33(66%)	20(40%)			33(67.4%)	18(36.7%)			31(62%)	18(36%)			34(68%)	21(42%)			166(66.7%)	100(40.2%)		
FPG, mmol/L	9.7±2.4	9.3±2.8	-0.3±2.7	0.374	9.6±2.5	9.0±3.3	-0.7±3.0	0.117	9.6±3.0	9.3±3.2	-0.4±3.3	0.442	9.7±3.0	8.7±2.5	-1.0±3.1	0.037	9.6±2.6	9.5±4.3	-0.2±4.7	0.804	9.7±2.7	9.2±3.3	-0.5±3.4	0.021
Weight, kg	80.2±12.4	78.7±12.2	-1.5±5.0	0.037	80.1±12.7	78.0±11.7	-2.1±3.4	<0.001	80.4±12.5	78.2±11.7	-2.2±4.1	<0.001	80.7±10.9	78.4±10.5	-2.2±3.2	<0.001	80.2±11.7	78.1±11.3	-2.1±3.0	<0.001	80.3±12.0	78.28±11.4	-2.0±3.8	<0.001
SBP, mmHg	142.5±17.6	141.1±17.2	-1.4±10.8	0.377	142.7±18.8	140.4±17.3	-2.4±6.5	0.012	142.9±18.0	140.9±15.6	-2.0±5.6	0.016	142.9±18.9	139.7±17.2	-3.2±6.5	<0.001	142.8±17.4	139.8±16.7	-2.9±4.9	<0.001	142.7±18.0	140.4±16.7	-2.4±7.1	<0.001
DBP, mmHg	82.5±8.1	82.1±8.9	-0.4±7.1	0.678	82.9±10.2	83.2±9.8	0.2±7.2	0.814	81.0±10.1	80.5±10.3	-0.5±8.0	0.682	82.4±9.1	81.4±9.1	-1.0±6.0	0.241	80.3±11.2	80.7±11.6	0.38±5.7	0.638	81.8±9.8	81.6±10.0.	-0.3±6.8	0.557

Data are presented as n (%) for categorical variables and as the mean ± SD for continuous variables
Exenatide-IR exenatide immediate-release, FPG fasting plasma glucose, SBP systolic blood pressure, DBP diastolic blood pressure
